# Potential role of ^68^Ga- and ^177^Lu-cyclic pentapeptides for in-vivo targeting CXCR4 receptor expression in chemotherapy relapse MCL patient

**DOI:** 10.22038/aojnmb.2025.85362.1615

**Published:** 2026

**Authors:** Tamanna Lakhanpal, Bhagwant Rai Mittal, Jaya Shukla, Yogesh Rathore, Rajender Kumar, Harmadeep Singh, Nivedita Rana

**Affiliations:** Department of Nuclear Medicine & PET Centre, Post Graduate Institute of Medical Education and Research, Chandigarh, India

**Keywords:** Keywords: Theragnostics, Cyclic pentapeptides, PET-CT, Lymphoma Immunophenotyping

## Abstract

**Objective(s):**

High levels of CXCR4 expression in patients with mantle cell lymphoma is associated with poor prognosis. Various molecular techniques used are unable to specify the metastatic disease burden. Cyclic pentapeptides act as CXCR4 antagonists hence are functional markers of in-vivo CXCR4 receptor expression. In this view, the theragnostic complex of radiolabeled ^68^Ga- and ^177^Lu-cyclic pentapeptides was developed to in-vivo target the CXCR4 receptor expression.

**Methods:**

Bone marrow aspiration and flow cytometry were performed to examine the fraction of lymphoid cells and immunophenotyping respectively. In-vitro CXCR4 receptor expression in the biopsied sample was determined using immunohistochemistry and flow cytometry molecular techniques. Diagnostic imaging using ^68^Ga-cyclic pentapeptide was performed to check the in-vivo CXCR4 expression in chemotherapy relapse MCL patient. Dosimetry studies in the same patient was performed with different time-point imaging to calculate the residence time and predict the critical organ.

**Results:**

Bone marrow aspiration indicated ~75% atypical lymphoid cells. Flow cytometric immunophenotyping revealed positivity for CD19, CD20, CD79b, Anti-kappa markers. IHC results showed high nuclear positivity. Approximately 86.11% of the cell population showed CXCR4 positive expression. Diagnostic imaging using ^68^Ga-cyclic pentapeptide showed high tracer avidity in the mesenteric mass at L4 level. The avidity of both ^68^Ga- and ^177^Lu- cyclic pentapeptide radiotracers was noted in the mesenteric mass at the L4 level. Dosimetry study using ^177^Lu-cyclic pentapeptide indicated kidneys as the critical organ with max residence time of 5.39 h.

**Conclusion:**

Theragnostic complex of radiolabelled ^68^Ga/^177^Lu- cyclic pentapeptides have the potential to in-vivo target the CXCR4 receptor expression.

## Introduction

 CXCR4 expression plays a pivotal role in the leukocyte infiltration, angiogenesis and cancer metastasis. CXCR4 receptor expression is generally assessed using tissue biopsy sampling, IHC (immunohistochemistry), and FC (flow cytometry). However, these techniques are not the accurate representation of primary or metastatic disease burden. This hiatus is fulfilled using tumor receptor imaging with radiolabeled ^68^Ga-and ^177^Lu-cyclic pentapeptides which provides the landscape of receptor expression, thereby, evaluating the entire tumor burden, as well as in the characterization of the heterogenicity of in-vivo tumor receptor expression in a non-invasive manner. 

## Methods

**Figure 1:**
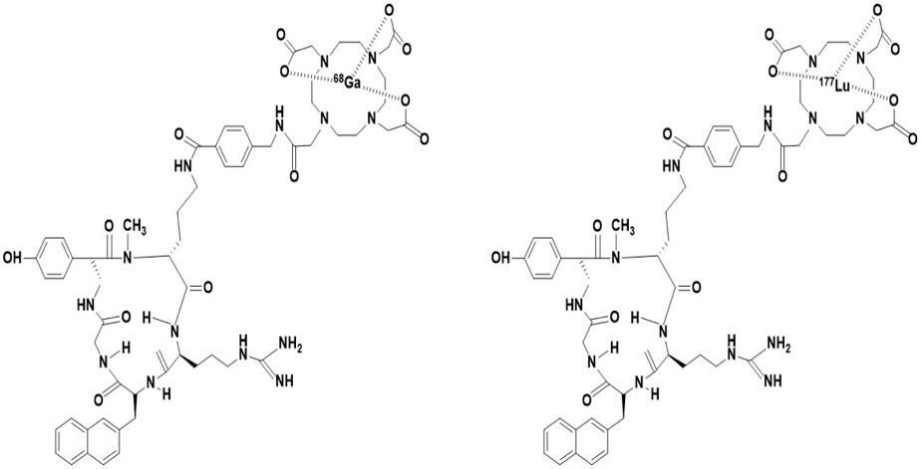
The chemical structures of in-house synthesized ^68^Ga- and ^177^Lu-cyclic pentapeptide CXCR4 antagonists

 The chemical structure of cyclic pentapeptide CXCR4 antagonist molecule is shown in Figure 1. The radiolabelling of commercially available CXCR4 antagonist, a cyclic pentapeptide was optimized with ^68^Ga (PET radionuclide) and ^177^Lu (SPECT radionuclide) for varying parameters such as pH ranges (3-8), buffers (CH_3_COONH_4_, CH_3_COONa), temperature ranges (25-100 °C), incubation time (5-60 min) and maximum reaction volume (0.5-4 mL).

^68^Ga radionuclide was freshly eluted from ^68^Ge/^68^Ga generator in 4 mL 0.05 M hydrochloric acid (1N). While ^177^Lu radionuclide was procured from the Bhabha Atomic Research Center (BARC) Trombay, in 0.05 M hydrochloric acid. Specific activity (mCi/μg) and amount of activity (mCi) of the day were calculated at each time of procurement based on the specification sheet provided having the date, time and amount of dispensing radioactivity.

### Radiolabelling of Cyclic pentapeptide with 68Ga

 The freshly eluted ^68^GaCl_3_ in HCl (0.05 M, pH 1-2) from ^68^Ge/^68^Ga generator was subjected to radiolabelling with cyclic pentapeptide molecule. The radiolabelling yield of non-purified ^68^Ga-cyclic pentapeptide obtained was 80-85% using sodium acetate buffer (CH_3_COONa, 0.25 M, pH=8-9) at the pH adjusted to 4-4.5 (titration volume <1 mL) for incubation period 10-15 min at the reaction temperature 90-100 °C for ≤3 mL reaction volume. While, the maximum radiolabelling yield obtained using ammonium acetate buffer (CH_3_COONH_4_, 0.5 M, pH=5.5) was ≤30% at the same reaction parameters. The peptide concentration optimized for chemical reaction was 15-20 μg/synthesis. The radiolabelled ^68^Ga-cyclic pentapeptide was subjected to purification using Sep-Pak C-18 cartridges to achieve the radiochemical purity ≥99%.

### Radiolabeling of Cyclic pentapeptide with 177Lu (Dosimetry use)

The radiolabelling of cyclic pentapeptide was optimized with carrier-added ^177^Lu radio-nuclide. The quality of carrier-added ^177^Lu depends upon the specific activity (SA) depicted in mCi/μg at the present date. All parameters were standardized for the radiolabelling of cyclic pentapeptide with ^177^Lu radionuclide (SA=10 to 23 mCi/μg). The radiolabelling yield were standardized ≥99 % by using ammonium acetate buffer (CH_3_COONH_4_, 0.5 M, pH=5.5). While the radiolabelling yield using sodium acetate buffer (CH_3_COONa, 0.25 M, pH=4.5) was ≥93-99 %. 

 The reaction pH was standardized 5.5 for ammonium acetate buffer and 4.5 for sodium acetate buffer out of various pH ranges. The incubation time standardized was 45-60 min, and the optimized temperature for obtaining the maximum radiolabelling yield (≥99 %) of cyclic pentapeptide with ^177^Lu radionuclide was 90 to 100 °C. The reaction volume standardized was ≤2 mL for obtaining ≥99 % yield of radiolabelled ^177^Lu-cyclic pentapeptide. Radiolabelling purity ≤95% considered for the purification with C-18 Sep-Pak cartridges to achieve the radiochemical purity ≥99%.

 The patient consented to participate in a pilot study (IEC-11/2019-1412) conducted to evaluate the theragnostic potential of radiolabeled cyclic pentapeptides in chemotherapy refractory patients with non-Hodgkin’s lymphoma. A 52-year-old male, case of relapse/refractory MCL, post 6 cycles of R-CHOP presented with abdominal pain, loss of appetite, weight loss for past three months. 

 Bone marrow aspiration/Trephine biopsy indicated ~75% atypical lymphoid cells, mild anisopoikilocytosis, microcytic lymphochromic RBCs with a few elliptocytes and occasional ovalocytes, and abundant platelets. 

**Figure 2:**
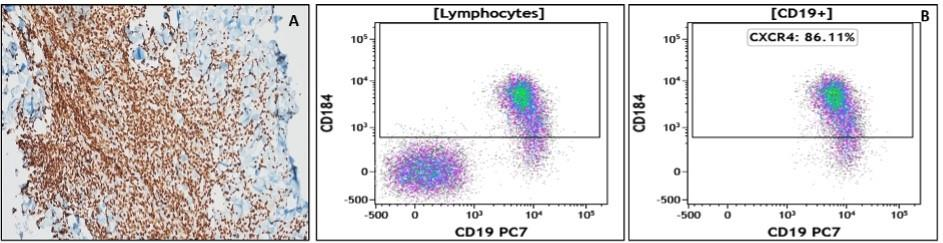
CXCR4 receptor expression determined using molecular techniques (**A**) immunohistochemistry and (**B**) flow cytometry

 Flowcytometric immunophenotyping revealed positivity for CD19, CD20, CD79b, Anti-kappa markers (Figure 2). The investigations showed impression of mature B-cell neoplasm.

## Results

**Figure 3:**
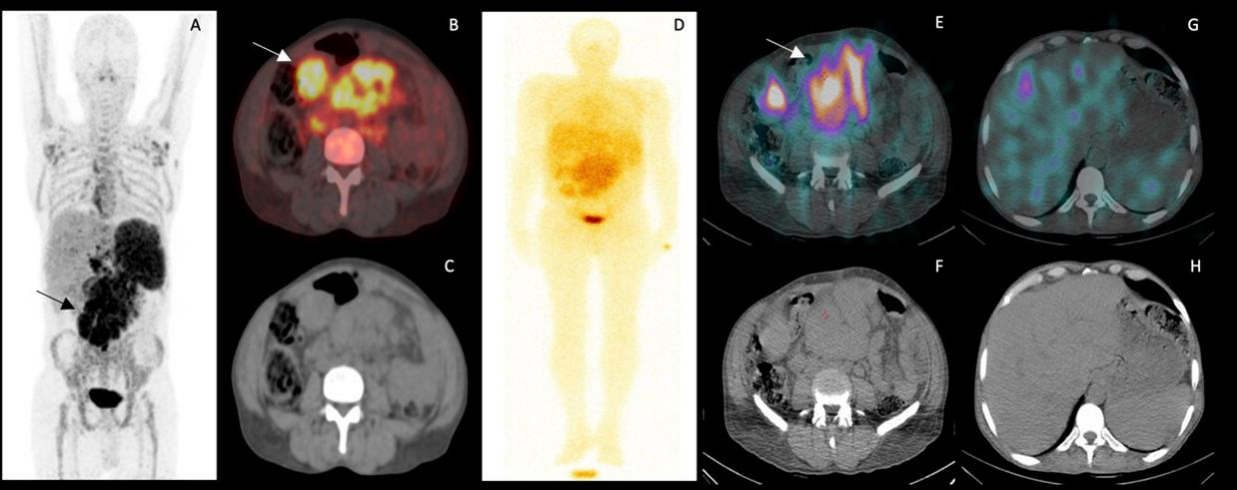
A 52yr/Male, c/o RR-MCL, post 6 cycles of R-CHOP underwent ^68^Ga-Cyclic pentapeptide (**A**-**C**) and ^177^Lu-Cyclic pentapeptide (**D**-**H**) imaging. MIP image of ^68^Ga- Cyclic pentapeptide (**A**) showed tracer uptake in the mesenteric mass and corresponding fused PET/CT (**B**) and CT images (**C**) of the abdomen showed high tracer avidity in the mesenteric mass at L4 level (SUV_max_=22.4, **arrow**). Whole-body planar images were acquired post intravenous administration of 473.6 MBq^177^Lu-Cyclic pentapeptide in the same patient up to 3 days for dosimetry purpose (Image **D**, Day 1). Axial fused SPECT/CT and corresponding CT images of the abdomen (**E** & **F**), showed high ^177^Lu-Cyclic pentapeptide avidity in the mesenteric mass (**arrow**). Physiological uptake of ^177^Lu-Cyclic pentapeptide was noted in the liver, spleen, kidneys, and high blood pool clearance. SPECT/CT image (**E**) was acquired at 24 h post radiotracer injection. In addition, faster clearance from hepatic tissues was noted at 24 h in the fused SPECT/CT imaging (**G**) and corresponding CT images (**H**)

 CXCR4 receptor expression in the biopsied sample was determined using two molecular techniques: IHC and FC (Figure 2). The IHC of the biopsied sample indicated intense CXCR4 positive nucleated cells (Intensity 3+). In FC results 86.11% cells population showed CXCR4 positive expression. In-vivo molecular imaging was performed using radiolabeled ^68^Ga- and ^177^Lu-cyclic pentapeptides. Initially, in-house synthesis of ^68^Ga-cyclic pentapeptide was carried out followed by all the quality control checks which included: radionuclide, radiochemical purity, sterility, and pyrogenicity. After all the quality control checks, the ^68^Ga-cyclic pentapeptide (88.8 MBq) radiopharmaceutical was intravenously administered to the patient followed by PET/CT imaging. To determine the therapeutic potential of ^177^Lu-cyclic pentapeptide, dosimetry studies were performed using OLINDA/EXM 2.0 computed software. For dosimetry studies whole-body planar imaging of the patient was performed up to 3-5 days, followed by a regional SPECT/CT at 24 h. The residence time (h) calculated was maximum for kidneys (5.39 h) followed by liver (4.84 h), and spleen (1.77 h). The critical organ determined was kidneys however, the radiotracer showed favourable pharmacokinetics and faster clearance from kidneys and hepatic tissues. Both the radiopharmaceuticals showed avidity in the mesenteric mass at L4 level (Figure 3).

## Discussion

 A number of studies have been reported in the literature to support the pattern of nuclear CXCR4 expression in various malignancies (1-4). In a study conducted by Yoshitake N et al (2008) that 29 out of 47 patients (61.7 %) with colorectal cancer (CRC), showed clear CXCR4 immunoreactivity in the nucleus and a weak signal was detected in the cytoplasm (nuclear type CXCR4 receptor expression)(1-4). It has been reported in literature that there may be variable uptake in the organs showing physiological biodistribution determined by the CXCR4 receptor expression in them (5). 

 Furthermore, the dosimetry studies are based on time-point imaging (pre-void, post-void imaging at 0 h and 24 h, regional SPECT-CT), day2 (48 h); and day 3/5). Initially, the uptake in the blood-pool; spleen, liver and bone marrow tend to be specifically higher than the lesion. It has been noted that with serial time-point imaging, the uptake in the lesion subsequently increases, the maximum uptake was noted at 24 h post radiotracer administration (max target to background ratio) concordant to the one indicated in regional SPECT-CT (represented as E and G in Figure 3).

 In addition, various molecular probes have been developed so far to target the CXCR4 receptor expression (6-9). We are presently working for the development of newer CXCR4 antagonists to target the CXCR4 expression, in this context some of the preliminary work has been done (10).

## Conclusion

 The strong positive correlation was found between the ^68^Ga /^177^Lu- cyclic pentapeptide uptake, and IHC and FC. Radiolabelled ^68^Ga- and ^177^Lu- cyclic pentapeptides has the potential to target CXCR4 receptor expression in patients with MCL. Kidneys were the critical organ determined using dosimetry. 
